# Complexin in ivermectin resistance in body lice

**DOI:** 10.1371/journal.pgen.1007569

**Published:** 2018-08-06

**Authors:** Nadia Amanzougaghene, Florence Fenollar, Claude Nappez, Amira Ben-Amara, Philippe Decloquement, Said Azza, Yassina Bechah, Eric Chabrière, Didier Raoult, Oleg Mediannikov

**Affiliations:** 1 Aix Marseille Univ, IRD, APHM, MEPHI, IHU-Méditerranée Infection, Marseille, France; 2 Aix Marseille Univ, IRD, APHM, VITROME, IHU-Méditerranée Infection, Marseille, France; Fred Hutchinson Cancer Research Center, UNITED STATES

## Abstract

Ivermectin has emerged as very promising pediculicide, particularly in cases of resistance to commonly used pediculicides. Recently, however, the first field-evolved ivermectin-resistance in lice was reported. To gain insight into the mechanisms underlying ivermectin-resistance, we both looked for mutations in the ivermectin-target site (GluCl) and searched the entire proteome for potential new loci involved in resistance from laboratory susceptible and ivermectin-selected resistant body lice. Polymorphism analysis of cDNA GluCl showed no non-silent mutations. Proteomic analysis identified 22 differentially regulated proteins, of which 13 were upregulated and 9 were downregulated in the resistant strain. We evaluated the correlation between mRNA and protein levels by qRT-PCR and found that the trend in transcriptional variation was consistent with the proteomic changes. Among differentially expressed proteins, a complexin i.e. a neuronal protein which plays a key role in regulating neurotransmitter release, was shown to be the most significantly down-expressed in the ivermectin-resistant lice. Moreover, DNA-mutation analysis revealed that some complexin transcripts from resistant lice gained a premature stop codon, suggesting that this down-expression might be due, in part, to secondary effects of a nonsense mutation inside the gene. We further confirmed the association between complexin and ivermectin-resistance by RNA-interfering and found that knocking down the complexin expression induces resistance to ivermectin in susceptible lice. Our results provide evidence that complexin plays a significant role in regulating ivermectin resistance in body lice and represents the first evidence that links complexin to insecticide resistance.

## Author summary

Through its unique mode of action, ivermectin represents a relatively new and very promising tool to combat multiple helminthic diseases and other infestations in humans and animals. However, ivermectin resistance in the field began to be reported. Therefore, understanding the mechanisms involved is a key step in delaying and tackling this phenomenon. In this study, through proteomic analysis of laboratory susceptible and ivermectin-selected resistant body lice, a complexin, a neuronal protein that plays a key role in regulating neurotransmitter release, was shown to be the most significantly down-expressed protein in ivermectin-resistant lice. Its down-expression by RNA-interference in susceptible lice induced resistance to ivermectin, providing evidence that complexin plays a significant role in regulating ivermectin resistance. This is the first evidence linking complexin to insecticide resistance.

## Introduction

Sucking lice (*Anoplura*) are obligate blood-feeding ectoparasites of eutherian mammals [1]. Humans are the preferred host for two species: *Pthirus pubis* and *Pediculus humanus* [2,3]. The latter has significant relevance to public health and includes two ecotypes: head lice (*P*. *h*. *capitis*), which live in the hair, and body lice (*P*. *h*. *humanus*), which live in clothing [1,3,4]. Head lice are common and can be found worldwide [1], with children being at increased risk [2]. Conversely, body lice are associated with poor socio-economic conditions [1,4] and homeless people and refugee-camp populations are predominantly affected [4,5].

Body lice are the main vectors of at least three dangerous pathogenic bacteria, namely: *Rickettsia prowazekii*, *Bartonella quintana* and *Borrelia recurrentis* [1,4]. The prevalence of the body louse is underestimated in many developed countries and, as the number of homeless people increases, louse-borne infectious diseases are also on the rise [1,5]. Recently, more emphasis has been placed on the ability of head lice to transmit bacterial diseases. Indeed, the DNA of several pathogenic bacteria is being increasingly detected in head lice, such as: *B*. *quintana*, *B*. *recurrentis* and *Yersinia pestis* [6–8]. In addition, studies have shown that experimentally infected head lice are capable of acquiring, maintaining and transmitting *R*. *prowazekii* and *B*. *quintana*, demonstrating that these lice have the potential to be a vector of pathogen under optimal epidemiological conditions [9,10]. This fact may pose a very substantial threat to humanity, as such infestations are not controlled in any country, including developed countries, despite repeated efforts to eradicate them [11].

This is mainly due to the resistance developed by lice to widely-used insecticides such as malathion and pyrethroid [1,12]. The use of new effective products with different modes of action, such as ivermectin (IVM), have proven to be a promising alternative to combating the problem of resistance [1,13]

IVM belongs to the macrocyclic lactone complex [14,15] and blocks synaptic transmission in invertebrates by binding to glutamate-gated chlorine channels (GluCls) in nerves and muscles, which are its primary target, leading to hyperpolarization, paralysis and death [14]. GluCls are not present in vertebrates and, as such, are thought to confer the broad safety margin of IVM [15]. IVM was the world’s first endectocide, capable of killing a wide variety of parasites and vectors, including lice [16]. IVM is already used to treat human lice and several reports indicated that both orally and topically formulations were highly effective in controlling lice infestations [13,17–20]. Moreover, the study conducted by Sangaré et al, showed that combination therapy with doxycycline plus IVM was highly effective compared to IVM alone in treating and preventing body lice under laboratory conditions and could be used to completely eradicate lice and potentially delay the emergence of IVM resistance [21].

Currently, resistance to IVM has been widely demonstrated in many arthropods and is an increasing problem for their control [22,23]. Recently, field evolved resistance to oral IVM treatment in head lice was documented in Senegal, for the first time, and was reported to cause reduced field control efficacy [24]. Understanding the mechanisms of IVM resistance is, therefore, a key step in delaying and tackling this phenomenon. IVM resistance in arthropods has been associated with several mechanisms, including reduced cuticular penetration [22], mutation in the target site [25] and metabolic resistance due to the overexpression of xenobiotic pumps from the ABC family [22,23,26]. Although mechanisms of IVM-resistance in lice have not yet been elucidated, in an attempt to identify inducible metabolic factors involved in IVM-tolerance, Yoon *et al*., showed that IVM induced detoxification genes, including ATP binding cassette and cytochrome P450, suggesting their association with its xenobiotic metabolism, thereby resulting in tolerance [27].

To gain a deeper understanding of mechanisms underlying IVM resistance in lice, we analyzed the IVM-target site from laboratory susceptible and IVM-selected resistant strains. Additionally, we used functional proteomics and performed a global proteomic analysis between the two strains. In addition, we assessed the correlation between mRNA and protein levels for differentially expressed proteins using quantitative real-time PCR, and further verified the functionality of a key candidate gene by RNA interference (RNAi).

## Results

### Resistance levels of selected laboratory lice against IVM

From the body lice susceptible strain (Orlando strain; Lab-IVS), IVM resistant selection (Lab-IVR) was successfully achieved by continuous exposure to IVM for ten generations in the laboratory. The median lethal time (LT_50_) value for Lab-IVS strain was 28.83 hours (24.47–32.78 hours), and for the Lab-IVR strain, 157 hours (144.91–172.37) (Table 1, S4 Table). The Lab-IVR strain exhibited 5.4-fold greater resistance against IVM when compared with the reference Lab-IVS strain, suggesting that the Lab-IVR strain had developed low and moderate resistance.

**Table 1:** Resistance of the selected strain (Lab-IVR) to IVM based on a comparison of median lethal (LT_50_) at a dose of 150 μg/Kg.

Lice	N	LT_50_ (hours) (95% CL)	Slope ± SE	χ2	RR
Lab-IVS	500	28.83 (24.47–32.78)	4 ± 0.85	10	-
Lab-IVR (selected)	300	157.01 (144.91–172.37)	0.86± 0.061	3.4	5.4

CL: Confidence limited; SE: Standard error; RR: Resistance ratio calculated by dividing the LT_50_ of the Lab-IVR strain by LT_50_ of Lab-IVS strain

### Cloning the body louse cDNA GluCl and polymorphism analysis

The open reading frame (ORF) of the body louse GluCl was composed of 1,110 nucleotides encoding 369 amino acids. Analysis of the polymorphism patterns of the cDNA sequences from the Lab-IVR (48 sequences) and Lab-IVS (48 sequences) strains showed the presence of six-point synonymous mutations thymine to cytosine (T363C), thymine to cytosine (T385C), guanine to adenine (G417A), guanine to adenine (G447A), adenine to guanine (A594G) and cytosine to thymine (C897T) (numbering based on the reference sequence XM002429761; S5 Table). The G417A and C897T mutations were found in both Lab-IVR and Lab-IVS strains, while the remaining four mutations were specific to the Lab-IVR strain.

### Identification of differentially expressed proteins between IVM-resistant and susceptible lice

Differentially expressed proteins from the Lab-IVS and Lab-IVR strains were identified and quantified by label-free Nano-LC-MS/MS analysis. In total, 407 proteins were identified, including 22 which were differentially expressed, of which 13 were up-regulated and 9 were down-regulated in the resistant Lab-IVR strain (Table 2). Gene ontology annotation, including molecular function, biological process and cellular component, was conducted to categorize these proteins (Table 3 and S1 Fig). The main molecular functions were catalytic activity and binding for both down- and up-regulated proteins. The cellular components of down- and up-regulated proteins were mainly cell, membrane, organelle and macromolecular complex. According on biological process, the proteins were mainly classified in cellular process, single-organism process and metabolic process (S1 Fig and S2 Fig).

**Table 2:** Differentially expressed proteins identified by proteomic analysis of the IVM-resistant strain compared to the susceptible strain.

Acc. ID	Description	Peptide count	Unique Peptide	Anova (*p*)	Fold change
**Up-regulated**					
E0W486	Adenylate kinase	16	15	0.000	3.08
E0VX56	ATP synthase delta chain, putative	11	11	0.005	2.23
E0W1N3	Heavy-chain filboin, putative	6	6	0.006	2.51
E0VW06	Mitochondrial outer membrane porin channel	28	25	0.009	2.12
E0VGH4	Limpet, putative	7	7	0.009	4.14
E0VSM7	Tubulin alpha-1 chain	75	19	0.015	3.05
E0VGF0	Putative uncharacterized protein	10	9	0.018	2.16
E0VQ79	Tubulin beta-2 chain, putative	17	8	0.024	2.31
E0W0W7	Putative uncharacterized protein	4	3	0.029	3.85
E0VYE0	Ornithine aminotransferase, putative	6	6	0.034	2.10
E0VHY3	Heat shock protein, putative	6	6	0.043	2.03
E0VSN4	Isocitrate dehydrogenase [NAD] subunit, mitochondrial	7	7	0.048	2.61
E0W2K0	Ejaculatory bulb-specific protein 3, putative	51	16	0.049	3.37
**Down-regulated**					
E0VJX5	Complexin, putative	3	3	0.000	-10.24
E0W229	Sodium/potassium-transporting ATPase subunit beta-2, putative	3	2	0.004	-2.97
E0VPU2	40S ribosomal protein S3a	3	2	0.004	-3.93
E0VWY3	D-beta-hydroxybutyrate dehydrogenase, putative	5	4	0.009	-2.68
E0VD43	Clathrin heavy chain	19	16	0.02	-3.24
E0VFA6	Trypsin	4	3	0.03	-3.78
E0VQK9	Guanine nucleotide-binding protein G(O) subunit alpha, putative	6	2	0.035	-2.85
E0VRZ6	Zinc finger protein CDGSH domain-containing protein, putative	3	3	0.035	-4.69
E0VKP4	Actin, muscle	164	61	0.05	-2.31

**Table 3:** Functional annotation of differentially expressed proteins.

Acc. No.	Gene name	GO analysis (Molecular function)	GO analysis (Biological process)	GO analysis (Cellular component)	InterPro (IP)	KEGG_PATHWAY
E0W486	Adenylate kinase, putative	Adenylate kinase activity, ATP binding,	ADP biosynthetic process, AMP metabolic process, ATP metabolic process,	mitochondrial intermembrane space, cytosol,	Adenylate kinase	Purine metabolism, Thiamine metabolism, Biosynthesis of antibiotics
E0VX56	ATP synthase delta chain, putative	proton-transporting ATPase activity, rotational mechanism	ATP synthesis coupled proton transport	proton-transporting ATP synthase complex, catalytic core	ATPase, F1 complex, delta/epsilon subunit	Oxidative phosphorylation, Purine metabolism, Thiamine metabolism
E0W1N3	Heavy-chain filboin, putative					
E0VW06	Mitochondrial outer membrane porin channel	voltage-gated anion channel activity	sperm individualization, transmembrane transport, regulation of cilium assembly, regulation of anion transmembrane transport, mitochondrial transport, photoreceptor cell maintenance	mitochondrial outer membrane, microtubule associated complex, lipid particle, nebenkern	Porin	
E0VGH4	limpet, putative	zinc ion binding			Zinc finger, LIM-type,	
E0VSM7	Tubulin alpha chain	GTP binding, GTPase activity, structural constituent of cytoskeleton	microtubule-based process	cytoplasm, microtubule		Purine metabolism, Thiamine metabolism
E0VGF0	Uncharacterized protein	methyltransferase activity, transferase activity	methylation		Farnesoic acid O-methyl transferase	
E0VQ79	Tubulin beta chain	GTP binding, GTPase activity, structural constituent of cytoskeleton	microtubule-based process	cytoplasm, microtubule		Purine metabolism, Thiamine metabolism
E0W0W7	Uncharacterized protein	carboxypeptidase activity	proteolysis	membrane, integral component of membrane	carboxypeptidase inhibitor	
E0VYE0	Ornithine aminotransferase	pyridoxal phosphate binding, identical protein binding, ornithine-oxo-acid transaminase activity	arginine catabolic process to glutamate, arginine catabolic process to proline via ornithine	cytoplasm		Arginine and proline metabolism, Metabolic pathways, Biosynthesis of antibiotics
E0VHY3	Heat shock protein, putative		Stress response			
E0VSN4	Isocitrate dehydrogenase [NAD] subunit, mitochondrial	magnesium ion binding, isocitrate dehydrogenase (NAD+) activity, NAD binding	tricarboxylic acid cycle	mitochondrion, integral component of membrane	Isocitrate and isopropylmalate dehydrogenases family	Citrate cycle (TCA cycle), Metabolic pathways, Biosynthesis of antibiotics, Carbon metabolism, 2-Oxocarboxylic acid metabolism, Biosynthesis of amino acids
E0W2K0	Ejaculatory bulb-specific protein 3 precursor				Insect pheromone-binding protein A10/OS-D,	
E0VJX5	**Complexin, putative**	syntaxin binding	neurotransmitter transport		Synaphin,	
E0W229	Sodium potassium-transporting ATPase subunit beta-2		potassium ion transport, sodium ion transport	sodium: potassium-exchanging ATPase complex		
E0VPU2	40S ribosomal protein S3a	structural constituent of ribosome	translation	cytosolic small ribosomal subunit	Ribosomal protein S3Ae	Ribosome
E0VWY3	D-beta-hydroxybutyrate dehydrogenase	retinol dehydrogenase activity	oxidation-reduction process	integral component of membrane	Glucose/ribitol dehydrogenase, NAD(P)-binding domain,	
E0VD43	Clathrin heavy chain	structural molecule activity	vesicle-mediated transport, intracellular protein transport	Clathrin coat of trans-Golgi network vesicle, Clathrin coat of coated pit		Lysosome, Endocytosis
E0VFA6	Trypsin	serine-type endopeptidase activity	proteolysis			Neuroactive ligand-receptor interaction
E0VQK9	Guanine nucleotide-binding protein G(O) subunit alpha,	GTP binding, G-protein coupled receptor binding, metal ion binding, GTPase activity, G-protein beta/gamma-subunit complex binding, signal transducer activity, metal ion binding	adenylate cyclase-modulating G-protein coupled receptor signaling pathway	heterotrimeric G-protein complex		
E0VRZ6	Zinc finger protein CDGSH domain-containing protein	2 iron, 2 sulfur cluster binding		intracellular membrane-bounded organelle, integral component of membrane		
E0VKP4	Actin, muscle	ATP binding, isopentenyl-diphosphate delta-isomerase activity, hydrolase activity	isoprenoid biosynthetic process			Biosynthesis of antibiotics, Terpenoid backbone biosynthesis

### Validation of proteomics data at the RNA level by qRT-PCR

**Fig 1:**
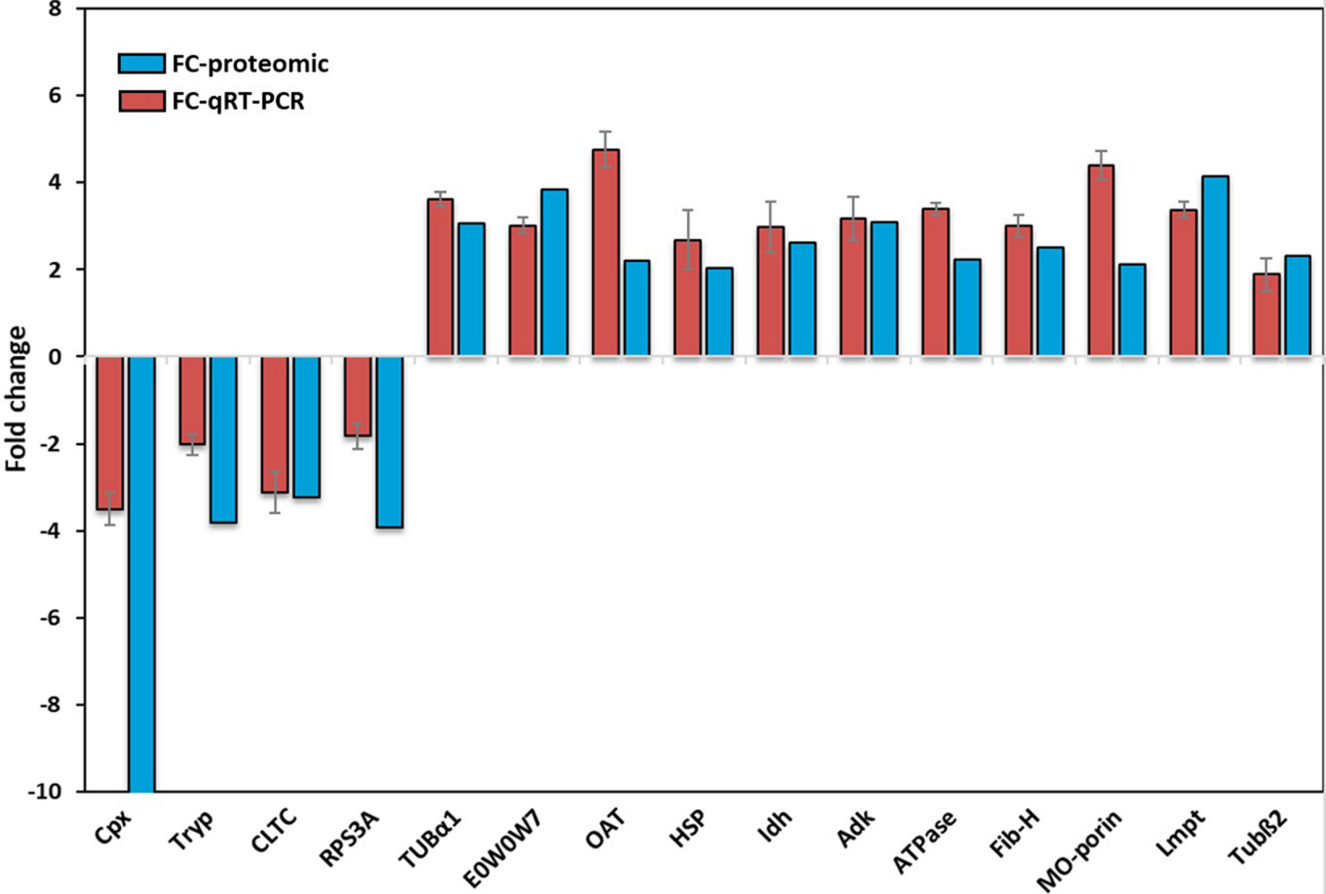
Comparison of proteomic and qRT-PCR results. The x-axis shows the 15 selected genes, while the y-axis gives the fold change observed for the Lab-IVR vs the Lab-IVS strains. EF1α was used to normalize the mRNA levels. Values are means ±SEMs (n = 3). The selected genes are: Cpx: Complexin, Tryp: Trypsin, CLTC: Clathrin heavy chain, RPS3A: 40S ribosomal protein S3a, TUBα1: Tubulinα1, OAT: Ornithine aminotransferase, HSP: Heat shock protein, Idh: Isocitrate dehydrogenase subunit, Adk: Adenylate kinase, ATPase: ATP synthase, Fib-H: Heavy-chain filboin, MO-porin: Mitochondrial outer membrane porin channel, Lmpt: Limpet, Tubß2: Tubulinß2 and E0W0W7.

To evaluate the proteomic data and the correlation between mRNA and protein levels, we randomly selected 15 differentially expressed genes to quantify their relative expression levels by qRT-PCR. As shown in Fig 1 and S6 Table, the trend in transcriptional variation for all the selected proteins was consistent with the proteomic changes determined in the proteomic analysis, suggesting that this method is a reliable way of identifying and quantifying differentially expressed proteins in lice.

Of all the differentially expressed proteins, complexin (Cpx) showed the most dramatically altered expression at proteomic level (10-fold down-regulated in Lab-IVR strain), which was correlated at the mRNA level with a slight difference (3.4-fold down-regulated in Lab-IVR strain). Furthermore, because of its impact on neuronal functions as the key regulators of neurotransmitter release [28] we suggest that this gene may play a significant role in regulating the IVM resistance mechanism. Thus, a Cpx was selected as our candidate gene for the subsequent functional verification.

### Characterization of complete Cpx cDNA and its relationship to other Cpxs

**Fig 2:**
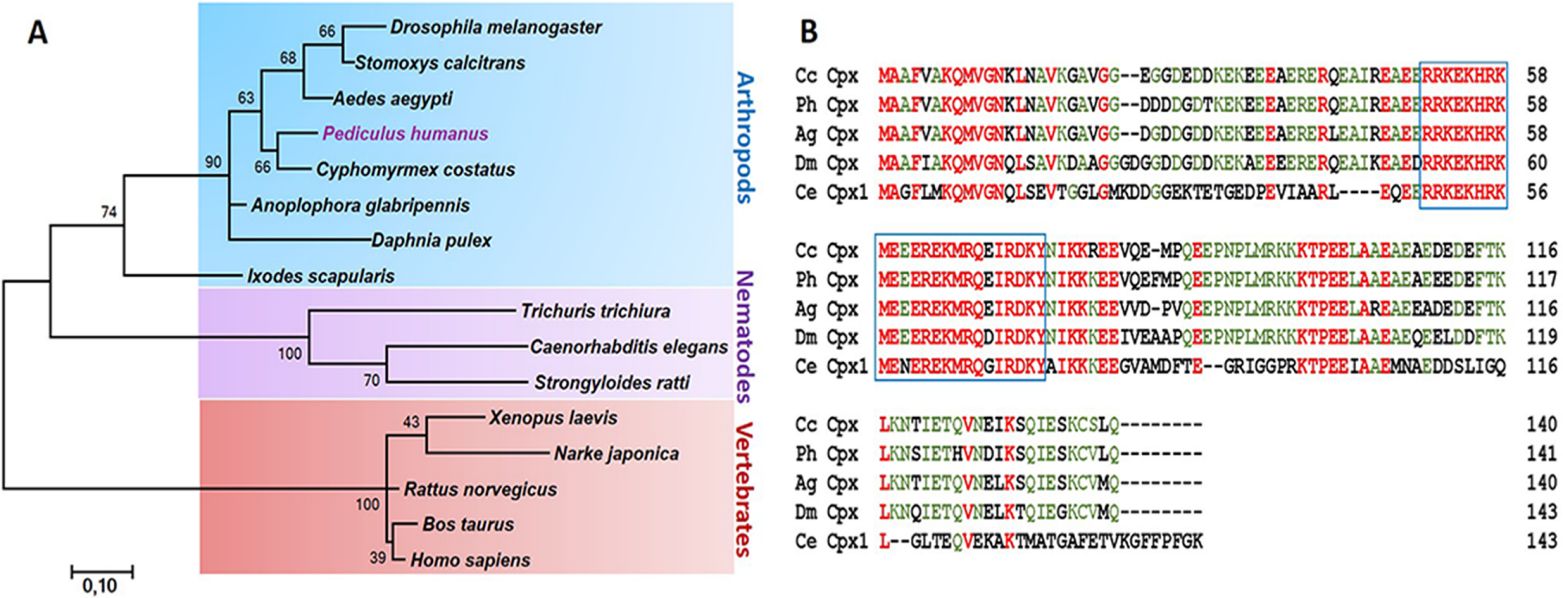
Comparison of Cpx proteins. (A) Phylogenetic tree showing the phylogenetic relationships of Cpx genes from Insecta and non-Insecta species. The tree was generated by ClustalW alignment of the amino acid sequences of Cpx genes using the neighbor-joining (NJ) method. (B) Amino acid sequence alignment of *P*. *humanus* (Ph) with members of the Cpx family. Identical residues are marked green and highly conserved residues are marked red. The blue box indicates the position of the predicted SNARE-binding domain. Dm: *Drosophila melanogaster*; Ce: *Caenorhabditis elegans*, Cc: *Cyphomyrmex costatus*, Ag: *Anoplophora glabripennis*.

The complete cDNA sequence of the Cpx was obtained by RT-PCR and RACE methods based on the partial sequence annotated from the body lice genome sequencing project. Its cDNA contains 426-bps open reading frame encoding 141 amino acids residues. A multiple sequence alignment of lice Cpx with insect and worms Cpxs demonstrates that homology is particularly high in the central predicted SNARE-binding domain (boxed) and in adjacent regions (Fig 2B, S7 Table). Homology analysis of amino acid sequence indicated that lice Cpx shared 91.5% identity with the ortholog in *Cyphomyrmex costatus*, 87.9% identity with *Anoplophora glabripennis*, 73.4% identity with *Drosophila melanogaster* and 44.7% identity with *Caenorhabditis elegans*. Phylogenetic relationships showed that lice Cpx clustered with insect Cpx and had the highest homology with *C*. *costatus* Cpx (Fig 2A).

### Cloning and polymorphism analysis of Cpx transcripts

To investigate whether mutations in the Cpx gene are involved in the mRNA downregulation leading to a decrease in protein expression observed in Lab-IVR, full-length ORF Cpx cDNA sequences were compared between the Lab-IVR and Lab-IVS strains. Cloning and sequencing (48 clones from each of the two independent cDNA batches) followed by multiple sequence comparison revealed the presence of a one nucleotide base pair insertion (A) at position 292-bps. The insertion was found on 17 clones out of 48 analyzed only from the Lab-IVR. This insertion causes a frameshift starting at amino acid 100 within the C-terminal domain of Cpx and results in a premature stop codon at amino acid 111 compared to normal Cpx (S3 Fig).

### Knockdown of Cpx gene by RNAi and subsequent decrease in the sensitivity of lice to IVM

**Fig 3:**
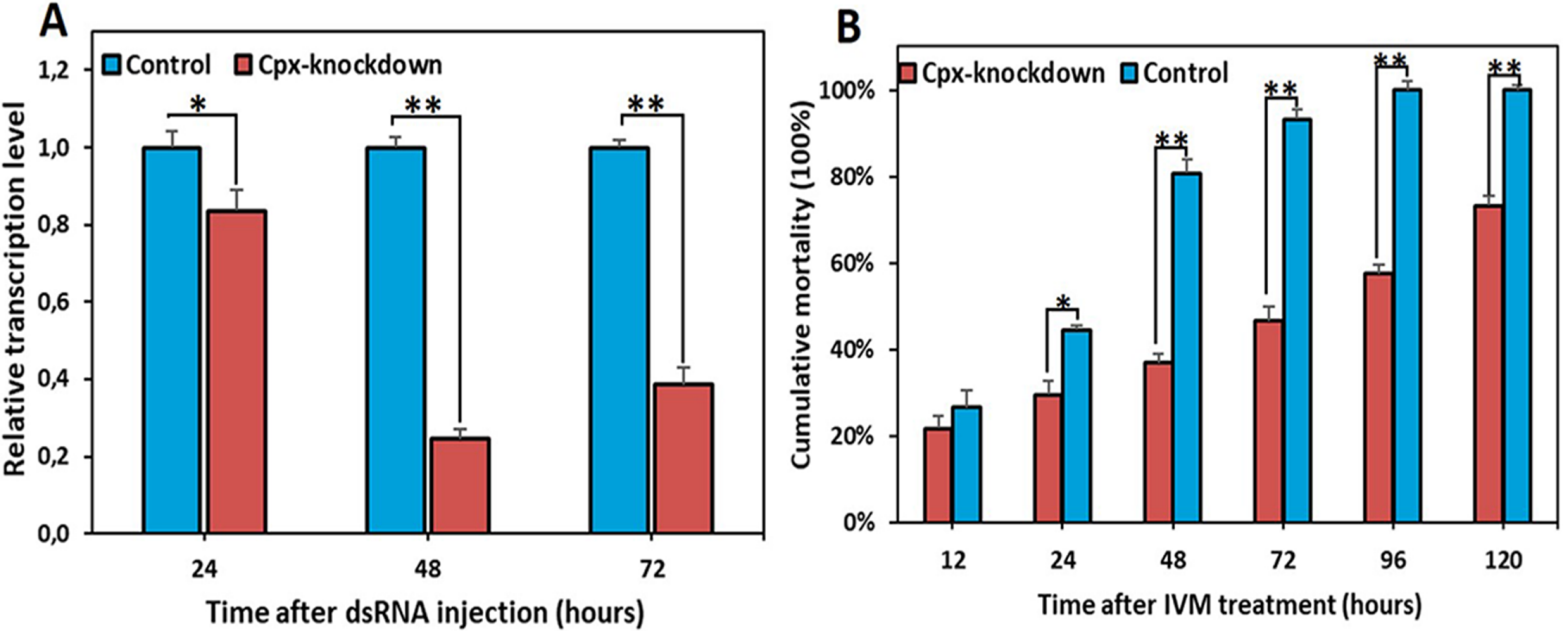
The effect of dsRNA on expression of Cpx in the IVM-susceptible strain and the effect of Cpx knockdown on IVM resistance. (A) Cpx mRNA levels were quantified by qRT-PCR in 72 hours of injection of dsRNA Cpx or pQE30 (control). The change in mRNA levels in the ds-RNA Cpx were calculated relative to controls. Values are means +SEMs (n = 3). Asterisks (*) indicate that Cpx dsRNA significantly suppresses the levels of Cpx transcript (*t-*test, *P<0.*05* and **P<0.*01*). (B) Bioassays for Lab-IVS lice exposed to IVM (150 μg/Kg) started 48 hours post-injection of dsRNA Cpx or pQE30 (control). Asterisks (*) indicate that knocking down Cpx expression decreased IVM-susceptibility in the Lab-IVS strain compared to the control (*Chi-2* test, *P<0.*05* and **P<0.*01*).

To further investigate the function of Cpx, we used RNAi technology to knockdown the expression of this gene in the susceptible Lab-IVS strain. The results showed that the Cpx mRNA levels reduced significantly, by 16.34% at 24 hours post-injection of dsRNA-Cpx and reached maximal reduction by 75.52% at 48 hours post-injection compared to the control injected with dsRNA-plasmid (pQE-30) (Fig 3A, S8 Table), indicating that this gene was mostly silenced by RNAi. No apparent physiological alterations were noticed in lice injected either with dsRNA Cpx or pQE-30 compared to the control non-injected lice. These findings demonstrate that dsRNA injection-based RNAi resulted in the knockdown of the Cpx gene in body lice.

To determine whether knocking down the expression of Cpx decrease the susceptibility to IVM in the susceptible Lab-IVS strain, we performed bioassays to compare IVM resistance levels among Lab-IVS lice at 48 hours post-injection with either dsRNA Cpx or pQE-30. The results showed that the mortality rates in lice down-expressing Cpx were significantly reduced compared to the control from 24 hours post exposure to a lethal dose of IVM (150 μg/kg) (*P* < 0.05, Fig 3B, S9 Table). Based on LT_50_, the IVM susceptibility of dsRNA-Cpx lice decreased by 3.2-fold (LT_50_ = 92.54 hours; Table 4) compared to the reference susceptible Lab-IVS (LT_50_ = 28.8 hours; Table 1) and it was only 2.2-fold higher than that in the Lab-IVR strain (LT_50_ = 157 hours, RR = 5.4; Table 1). Taken together, the RNAi-mediated knockdown of Cpx decreased the lice’s susceptibility to IVM (increased their resistance), providing relatively convincing evidence that this gene contributes to IVM resistance in this resistant strain.

**Table 4:** Comparison of median lethal time (LT_50_) between the lice with its Cpx knockdown (Cpx dsRNA-injected) and control lice (pQE30 dsRNA-injected).

Lice	N	LT_50_ (h) (95% CL)	Slope ± SE	χ2	RR
Cpx dsRNA-injected	165	92.54 (81.90–105.07)	0.78 ± 0.07	6.33	3.22
pQE30 dsRNA-injected	150	27.69 (24.31–30.82)	4± 0.11	1.94	0.96

RR: Resistance ratio calculated by dividing the LT_50_ of either the Cpx dsRNA or pQE30 dsRNA by LT_50_ of Lab-IVS

## Discussion

IVM is a very promising tool to fight different infestations such as, for instance, pediculosis, especially in cases of resistance to commonly used pediculicides. However, as with other insecticides, IVM is subject to selection pressures that have led to the development of resistance in many arthropods. To gain insight into the mechanisms underlying IVM resistance in body lice, we both looked for mutations in the GluCl and searched the entire proteome for potential new loci involved in resistance.

Firstly, the comparison of the cDNA GluCl between the Lab-IVR and Lab-IVS strains yielded no non-silent SNPs. This fact is most likely due to the lower level of resistance of our Lab-IVR strain. Indeed, in most published studies that have identified non-synonymous mutations in GluCl gene that affect IVM sensitivity, the level of resistance of the organism was considerably high. This is the case of *Plutella xylostella* where the A309V mutation was associated with 11 000-fold resistance [29], in *Tetranychus urticae* the G326E mutation was associated with 2000-fold resistance [30] and *D*. *melanogaster* where the P299S mutation was associated with more than 10-fold IVM resistance [25].

Although, target site insensitivity resulting from non-synonymous mutations within GluCl gene of the arthropod nervous system is known to be of primary importance in the development of resistance to IVM [25,30,31], the potential contribution of other types of mutations mediated by mechanisms other than changing protein sequences should not be ruled out. Indeed, recent research suggests that a variety of mechanisms involving changes to upstream regulators (change in trans), mutations of the noncoding regulatory DNA sequences (change in cis), as well as certain types of synonymous mutations of the coding sequences of a gene, may play a significant role in altering gene functions, including gene expression, formation of secondary structures of proteins, protein folding and substrate protein interaction [31–34]. In this study, six synonymous mutations were identified in the cDNA GluCl, of which four mutations were found only in the resistant Lab-IVR strain. Further studies are needed to examine the potential roles of these mutations in terms of protein folding and function.

Secondly, in our efforts to shed light on other possible mechanisms of resistance, we compared the overall protein expression pattern of the IVM-resistant and susceptible strains. In total, 22 proteins were differentially regulated, of which 13 were up-regulated and 9 were down-regulated in the resistant strain. Among the differentially expressed proteins found in the resistant strain, the most interesting observation was the activation of energy metabolism through the up-regulation of several key enzymes in the metabolic pathways (i.e. the isocitrate dehydrogenase [NAD] subunit, adenylate kinase, ATP synthase delta chain, tubulin alpha chain, tubulin beta chain, and ornithine aminotransferase), affecting the Krebs cycle, phosphorylation oxidative, purine, vitamins and amino acid metabolisms. Such nutrient availability may be necessary to overcome the elevated demands for energy and metabolism in the ‘toxic’ environment of the resistant lice, and consequently to maintain normal metabolism and energy balance at the cellular level. This is consistent with the fact that insecticide resistance is usually associated with higher demands for energy observed in other insect species [35,36].

A Cpx was the most significantly (P<0.0001) and highly downexpressed (10-fold) protein in the IVM-resistant lice. Indeed, this protein plays a key role in regulating synaptic exocytosis and neurotransmitter release. It was, therefore, selected to investigate its possible involvement in IVM-resistance. The Cpx cDNA from the body louse was identified and characterized. The deduced amino acid sequence presented very high similarity with the Cpx of other insects, suggesting that the basic mechanisms of its functions are similar to those described for *D*. *melanogaster*, the model organism, from which Cpx has been extensively studied.

The decrease of Cpx in protein expression was found to be associated with its mRNA downregulation, suggesting that the factors influencing Cpx expression occur at a pretranscriptional level. To gain insight into the mechanisms underlying this downexpression, we performed a DNA mutation analysis of Cpx transcripts and found that some mRNAs Cpx from the Lab-IVR strain had gained a premature stop codon. Therefore, the reduction of Cpx expression might be due, in part, to a secondary effect of a nonsense mutation inside the Cpx gene. Such a mechanism, known as nonsense-mediated mRNA decay, has been reported in insects [37,38] whereby mutations inside a gene cause premature termination codons and quickly degrade mRNA, inhibiting the accumulation of nonsense (inactive) proteins.

RNAi techniques for the suppression of specific transcripts is proving to be a powerful tool in several insect species [39]. Body louse genome analysis has been shown to contain the genes necessary for RNAi [40]. Subsequent studies have reported that the injection of dsRNA can effectively suppress target genes in lice, and this ability has been widely used in gene function research [27,41]. Thus, we evaluated the resistance function of Cpx in susceptible lice via RNAi. Our findings showed that the injection of dsRNA-Cpx resulted in an effective suppression of Cpx expression and significantly decreased susceptibility to IVM compared with the control. This result provides convincing evidence that Cpx plays an important role in conferring IVM resistance in the body louse studied and represents the first evidence linking Cpx to insecticide resistance.

Cpx is small neuron-specific cytosolic protein that interact with the assembled SNARE (soluble N ethylmaleimide-sensitive factor [NSF] attachment protein receptor) complex to modulate the vesicle fusion process and neurotransmitter release [28,42]. Despite intensive research, the precise functions of Cpx remain controversial and continue to present a conundrum [28,42]. For instance, genetic studies conducted in mammalians, worms and fruit flies have all shown that a Cpx has a dual function and can act either as inhibitory or facilitatory for neurotransmitter release depending on the species, type of synapse (at CNS or at neuromuscular junction (NMJ)) and whether or not the vesicles are activated by Ca2+ (spontaneous or evoked release) [28]. Although it is not known how this downregulated protein is associated with IVM resistance, as IVM acts as a ligand for the inhibitory ligand-gated ion channel, activated by its natural agonist neurotransmitter glutamate [15,43], we hypothesized that the effect of Cpx on IVM resistance may be through regulating the glutamate release machinery at glutamatergic synapses. Indeed, studies conducted on worms have shown that the affinity of IVM is enhanced dramatically in the presence of glutamate, suggesting that the natural ligand, by binding to a distinct site, can allosterically enhance the activity of IVM and exert complementary, and possibly additive, effects on the conformational changes needed for the channels to open [43–45]. Moreover, it was thought that the extraordinary potency of the IVM killing parasites at much lower concentrations than those needed to activate recombinant channels expressed in *Xenopus oocytes* [44] is due to the interaction between endogenous glutamate and IVM [45]. Taken together, one could speculate that the downexpression of Cpx constitutes a primary mechanism by which lice protect their CNS, given that the GluCls in insects are mostly expressed in CNS [46] and the function of Cpx in that synapses is speculated to be mostly facilitatory for neurotransmitter release [28].

Although many issues remain to be investigated, the result of our study is exciting and provides the first insights into the mechanism underlaying IVM resistance in body lice at proteomic level, and links Cpx to insecticide resistance for the first time.

## Materials and methods

### Ethics statement

Adult New Zealand white rabbits were obtained from Charles River laboratories, were handled according to the rules of N° 2013–118, February 7, 2013, France and the experimental protocols (references APAFIS # 01077.02 & 2015050417122619), were approved by the Ethics Committee “C2EA-14” of Aix-Marseilles University, France and the French Ministry of National Education, Higher Education and Research.

### Body louse populations

#### Body louse strain and rearing

The Orlando (Culpepper) reference strain of body lice, *P*. *h*. *humanus*, was used in this study. The lice were maintained at 29°C with 70%–90% humidity and fed three times a week on the shaved abdomen of anesthetized rabbits. This strain has been reared in the insectary of our laboratory for more than fifteen years without exposure to any insecticides and is used as a reference susceptible strain referred to as Lab-IVS.

#### Resistant population selection

The IVM resistant strain was selected from a susceptible strain with IVM, referred as Lab-IVR. The selection was performed by exposing each generation of adult lice to IVM for 30 minutes, by feeding them on a rabbit specifically dedicated to this purpose, that had received a subcutaneous injection of IVM (IVOMEC, Merial) three hours previously. The experiment started by exposing lice to an approximate median lethal dose (LD50) of 100 μg/kg of the rabbit’s body weight. This first exposure was carried out with three replicate groups, and each replicate group was initiated with more than 800 adult lice. All surviving lice were grouped together and were maintained without IVM exposure to obtain eggs for the next generation. The LD50 was determined through a preliminary assay conducted to generate dose response data (S1 and S2 Tables).

After three generations of selection at a dose of 100 μg/kg, the selected lice were then exposed to the constant concentration of 150 μg/kg. This dose killed 90 to 100% of susceptible Lab-IVS within 72 hours (S1 Table).

Selection was made over a seven-month period and mortality was maintained at about 20–30%. IVM susceptibility bioassays were initiated at the end of the selection and the number of dead and surviving lice was recorded after 12 hours. The LD50, the median lethal times (LT50) and 95% confidence intervals were calculated by probit analysis using the SPSS software (IBM software, Armonk, NY) [47].

### Comparison of proteomic profiles between IVM-resistant and susceptible lice

#### Protein preparation and digestion

Total protein was extracted from both IVM-resistant and susceptible lice (48-hours ten starved adult lice per sample, with four replicates of each). Samples were suspended in 200 μL of lysis buffer (8 M urea, 2 M thiourea, 100 mM NaCl, 25 Mm Tris, pH 8.2, complete protease inhibitor) and crushed with two 3-mm tungsten beads in TissueLyser at 25 Hz for four minutes (Qiagen, Courtaboeuf, France). After homogenization, the insoluble fractions were removed by centrifugation and soluble proteins were dialyzed twice using Slide-A-Lyzer MINI Dialysis Devices (Pierce Biotechnology, Rockford, USA) and dialysis buffer (50 mM ammonium bicarbonate, pH 7.4, 1 M Urea) following the manufacture’s protocol. Dialyzed fractions were collected, and protein concentration was determined by Bradford Protein Assay using Coomassie (Biorad, Marnes-la-Coquette, France). The dialyzed fractions were used as a template for global proteomic analysis. Briefly, 50 μg of total soluble proteins were reduced with 10 mM dithiothreitol for one hour at 30°C, alkylated with 20 mM iodoacetamide for one hour in the dark, and then digested by adding 2 μg of sequencing-grade trypsin solution (Promega, Charbonnières, France) for 20 hours at 37°C. The digested samples were then desalted using Pierce Detergent Removal Spin Columns (Thermo Fisher Scientific, Illkirch, France) following the manufacturer’s protocol.

#### Label-free quantitative nano-LC-MS/MS proteomics analysis

The protein digests were analyzed using a NanoAcquity UPLC System with two-dimensional liquid chromatography (2D-LC) Technology (Waters, Saint-Quentin-en-Yvelines, France) connected to a Synapt G2Si Q-TOF ion mobility hybrid mass spectrometer (TWIM-MS; Waters, Saint-Quentin-en-Yvelines, France). The first chromatographic dimension consisted of a 300-μm by 50-mm C18 column (Nano Ease 5 μm XBridge BEH130, Waters). Peptides were eluted onto a second dimension using a gradient of seven steps at 1.5 μl/min, with 20 mM ammonium formate pH 10, and 12, 15, 18, 20, 25, 35, and 65% acetonitrile. A trapping column (nanoAcquity UPLC 10K-2D-V/M Trap 5-μm Symmetry C18 column; 180 μm × 20 mm, Waters) was used to collect the first-dimension peptides for concentration and desalting, after dilution at 20 μl/min in 99.9% water–0.1% formic acid and 0.1% acetonitrile–0.1% formic acid. The second dimension consisted of a 75-μm by 250-mm C18 column (nanoAcquity UPLC 1.8-μm HSS T3; Waters). Peptides eluted from the first-dimension steps were separated using a 1-h gradient (275 nl/min; 5 to 40% acetonitrile–0.1% formic acid). Data-independent MS/MS analysis was performed with the ion mobility feature (HDMSe method). The ion source parameters were capillary voltage 3 kV, sampling cone voltage 40 V, ion source temperature 90°C, cone gas flow 50 L/h. Transfer collision low energy was set to 5 V and trap collision low energy was set to 4 V. The high energy ramp was applied from 4 V to 5 V for the trap collision and from 19 V to 45 V for the transfer collision enabling fragmentation of the ions after the ion mobility cell and before the time-of-flight (TOF) MS. On-column sample load was 285 ng per fraction (2 μl injected).

#### Data processing and analysis

The acquired files were imported into Progenesis QI software Version 2.0 (Nonlinear Dynamics, Newcastle, UK) for label-free quantification analysis. The data were aligned automatically and normalized. Processing parameters were 150 counts for the low energy threshold, 30 counts for the elevated energy threshold. Databases used to identify peptides combined data from Phthiraptera (TrembL, 03/17/2016, 14,329 sequences) and the Oryctolagus (TrembL, 03/18/2016, 23,018 sequences). Search tolerance parameters were: peptide and fragment tolerance, 15 ppm, FDR < 1%; minimum ion matching requirements were three fragments per peptide, seven fragments per protein and two peptides per protein. The enzyme specificity was trypsin allowing one missed cleavage, carbamidomethyl of cysteine (fixed), oxidation of methionine (variable), carbamyl of lysine and N-terminal (variable). Protein normalization was performed according to the relative quantitation using non-conflicting peptides.

ANOVA tests were performed to determine the significance of changes between samples. A fold-change of >2 and a *p*-value <0.05 in at least two replicates were used as the thresholds to define differently expressed proteins. Gene ontology analysis for the differentially expressed proteins was carried out using Blast2GO (https://www.blast2go.com/blast2go-pro/download-b2g). The metabolic pathway analysis was conducted according to the Kyoto Encyclopedia of Genes and Genomes (KEGG) Pathway Database (http://www.genome.jp/kegg).

### Total RNA extraction and reverse transcriptase-quantitative real-time PCR analysis (qRT-PCR)

#### Isolation of RNA and cDNA synthesis

Total RNA from a pool of six 48-hours starved lice from both the IVM-resistant and susceptible strains was extracted using the RNeasy Mini kit (Qiagen) according to the manufacturer’s instructions. The quantity and quality of the RNA were assessed using a NanoDrop ND-1000 (Thermo Fisher Scientific). First-strand cDNA was synthesized using MMLV-RT kit (Invitrogen) with oligo (dT) as primer, according to the manufacturer’s protocol.

#### Quantification of mRNA expression by quantitative real time PCR (qPCR)

Primers for qPCRs were designed from all the selected genes using the free web Primer3 software, version 4.0 (http://frodo.wi.mit.edu/primer3/) and their sequences were listed in S3 Table. qPCRs were performed using a CFX96 Real-Time system (Bio-Rad Laboratories, Foster City, CA, USA) with LightCycler FastStart DNA Master SYBR Green I (Roche applied Science) in accordance with the manufacturer’s instructions. Three biological replicates, with three technical replications for each, were evaluated for each sample. We chose the housekeeping gene elongation factor 1-α (EF1α) for internal normalization [27]. The Fold-changes (FC) of target genes relative to EF1α were calculated according to the 2^−ΔΔCt^ method [48].

### Cloning and sequence analysis of the Cpx and GluCl transcripts

Rapid amplification of cDNA ends (RACE) with SMARTer RACE cDNA Amplification kits (Clontech, PaloAlto, CA, USA) was used to obtain the full-length cDNA of Cpx following the manufacturer’s protocol, using universal primers supplied in the kits and gene-specific primers (GSPs) designed based on the partial cDNA sequence annotated from the body lice genome sequencing project (GenBank accession XM002426374). Subsequently, the full-length cDNA of Cpx was generated using a specific primer pair designed based on the 5’and 3’end sequences of the putative Cpx mRNA. Full-length cDNA was subjected to bioinformatic analysis using an ORF (open reading frame) finder tool (http://www.ncbi.nlm.nih.gov/gorf/gorf.html). Subsequently, the complete ORF of Cpx was amplified using the same cDNA synthesized for qRT-PCR from both resistant and susceptible strains to perform DNA polymorphism analysis. Amplification of the ORF cDNA GluCl was also conducted, as described for the Cpx gene, using a set of primers designed based on the cDNA gene sequence available in the NCBI database (GenBank accession XM002429761). All primers used are listed in S3 Table.

PCRs amplifications were performed using a Peltier PTC-200 thermal cycler (MJ Research Inc., Watertown, MA, USA) with the Hotstar Taq-polymerase (Qiagen). The purified PCR products were ligated into a pGEMT-easy vector (Promega) and transformed into JM109 Competent Cells. The plasmid inserts were PCR amplified using a vector-specific primer (M13 forward and reverse primers) and subjected to sequencing using the Big Dye Terminator Cycle Sequencing Kit (Perkin Elmer Applied Biosystems, Foster City, CA) with an ABI automated sequencer (Applied Biosystems). The electropherograms were assembled using ChromasPro (ChromasPro 1.7, Technelysium Pty Ltd., Tewantin, Australia). Alignment of the nucleotide and amino-acid sequences was conducted using the ClustalW2 computer program (http://www.ebi.ac.uk/Tools/clustalw2/index.html) and phylogenetic trees were constructed with MEGA7.1.

### Functional validation of the role of Cpx gene in IVM-resistance by RNAi

#### dsRNA synthesis and gene knockdown

A Cpx transcription template from the cDNA of wild type lice was generated by PCR amplification using gene-specific primers with the T7 promoter element attached at their 5’ ends (S3 Table). The purified PCR product was then used as a template in the dsRNA synthesis reaction using the MEGAscript RNAi Kit (Invitrogen) according to the manufacturer’s instructions. The dsRNA quality was evaluated by gel electrophoresis and quantified with a NanoDrop ND-1000 (Thermo Fisher Scientific). The dsRNA of the *Escherichia coli* plasmid amplified from *pQE-30* vector (Qiagen) was prepared as described above and used as control. RNAi experiments were carried out by injecting ~120 ng of dsRNA-Cpx to adult lice in the ventral side between the second and third posterior abdominal segments, as described previously [27], with the FemtoJet 4i injector (Eppendorf, Germany), using self-pulled glass capillary needles (World Precision Instruments, Germany) under an Axio Zoom V16 microscope (Carl Zeiss S.A.S., France). The injected dose was determined empirically through preliminary experiments by determining concentrations and volumes of dsRNA resulting in maximum levels of target gene silencing that caused no mortality at various time post-injection. Lice were also injected with the dsRNA of *pQE-30* plasmid as control. Total RNA was extracted from injected lice and processed to cDNA synthesis and qPCRs analysis to evaluate the degree of target gene silencing as described above. The relative expression level of Cpx of each ds-Cpx sample relative to the controls was calculated according to the 2^−ΔΔCt^ method [48] after normalization with the EF1α gene. The Student’s *t-*test was used to assess the statistical significance using GraphPad Prism version 7.00 for Windows (GraphPad Software, La Jolla California USA, www.graphpad.com).

#### Mortality bioassays following dsRNA-Complexin injection

Two groups of lice from either the Cpx dsRNA-injected (165 lice) or pQE30 dsRNA-injected (150 lice), at 48 hours post-injection, were exposed to IVM by feeding them on a rabbit that had received a dose of 150 μg/kg of IVM. Mortality was assessed over six hours after the IVM-treatment to calculate the value of LT_50_ and RR, as described above. The *Chi-2* test was used to assess the statistical significance.

## Supporting information

S1 Fig Gene ontology (GO) analysis of the differentially expressed proteins. The proteins are grouped into three GO terms: cellular component, biological process and molecular function.
(TIF)

S2 Fig Gene ontology assignment of downregulated and upregulated proteins related to molecular function (MF), biological processes (BP) and cellular component (CC). (TIF)

S3 Fig Amino acid sequences alignment of body lice Cpx transcripts. The alignment shows a frameshift starting at amino acid 100 and a premature stop codon at amino acid 111 in the mutated transcript (from the resistant lice) compared to normal Cpx transcript.
(TIF)

S1 Table Survival of lice (Lab-IVS strain) exposed to different dose of IVM at different time intervals. A total of 500 lice were exposed for each IVM dose.
(DOC)

S2 Table Susceptibility of the Lab-IVS strain to IVM. Probit regression data for the relationship between dose of ivermectin and mortality at 72 hours.
(DOC)

S3 Table Primer sequences used in this study. (DOC)

S4 Table Survival of lice of the selected strain (Lab-IVR) and the susceptible strain (Lab-IVS) exposed to dose of 150 μg/kg of IVM at different time intervals. (DOC)

S5 Table The six synonymous mutations identified in cDNA GluCl gene from the susceptible and IVM-selected resistant strains. (DOC)

S6 Table Comparison of fold change of proteomic and qRT-PCR results for the 15 selected genes. (DOC)

S7 Table Cpx accession numbers from distinct species used for construction of phylogenetic tree. (DOC)

S8 Table The relative expression level of Cpx of each ds-Cpx sample relative to the controls calculated according to the 2−ΔΔCt method after normalization with the EF1α gene. (DOC)

S9 Table Survival of lice exposed to IVM (150 μg/Kg) started 48 hours post-injection of dsRNA Cpx or pQE30 (control) at different time intervals. (DOC)

## References

[1] Bonilla DL, Durden LA, Eremeeva ME, Dasch GA. The biology and taxonomy of head and body lice—implications for louse-borne disease prevention. PLoS Pathog. 2013;9: e1003724 10.1371/journal.ppat.1003724 24244157 PMC3828170

[2] Chosidow O. Scabies and pediculosis. The Lancet. 2000;355: 819–826. 10.1016/S0140-6736(99)09458-1 10711939

[3] Veracx A, Raoult D. Biology and genetics of human head and body lice. Trends Parasitol. 2012;28: 563–571. 10.1016/j.pt.2012.09.003 23069652

[4] Raoult D, Roux V. The body louse as a vector of reemerging human diseases. Clin Infect Dis Off Publ Infect Dis Soc Am. 1999;29: 888–911. 10.1086/520454 10589908

[5] Brouqui P. Arthropod-Borne Diseases Associated with Political and Social Disorder. Annu Rev Entomol. 2011;56: 357–374. 10.1146/annurev-ento-120709-144739 20822446

[6] Amanzougaghene N, Akiana J, Mongo Ndombe G, Davoust B, Nsana NS, Parra H-J, et al Head Lice of Pygmies Reveal the Presence of Relapsing Fever Borreliae in the Republic of Congo. PLoS Negl Trop Dis. 2016;10: e0005142 10.1371/journal.pntd.0005142 27911894 PMC5135033

[7] Amanzougaghene N, Fenollar F, Sangaré AK, Sissoko MS, Doumbo OK, Raoult D, et al Detection of bacterial pathogens including potential new species in human head lice from Mali. PLOS ONE. 2017;12: e0184621 10.1371/journal.pone.0184621 28931077 PMC5606924

[8] Piarroux R, Abedi AA, Shako J-C, Kebela B, Karhemere S, Diatta G, et al Plague epidemics and lice, Democratic Republic of the Congo. Emerg Infect Dis. 2013;19: 505–506. 10.3201/eid1903.121542 23750356 PMC3647677

[9] Kim JH, Previte DJ, Yoon KS, Murenzi E, Koehler JE, Pittendrigh BR, et al Comparison of the proliferation and excretion of Bartonella quintana between body and head lice following oral challenge. Insect Mol Biol. 2017;26: 266–276. 10.1111/imb.12292 28105732 PMC5400725

[10] Murray ES, Torrey SB. Virulence of Rickettsia prowazeki for head lice. Ann N Y Acad Sci. 1975;266: 25–34. 829471 10.1111/j.1749-6632.1975.tb35086.x

[11] Raoult D, Abat C. Developing new insecticides to prevent chaos: the real future threat. Lancet Infect Dis. 2017;17: 804–805. 10.1016/S1473-3099(17)30395-X 28741543

[12] Durand R, Bouvresse S, Berdjane Z, Izri A, Chosidow O, Clark JM. Insecticide resistance in head lice: clinical, parasitological and genetic aspects. Clin Microbiol Infect Off Publ Eur Soc Clin Microbiol Infect Dis. 2012;18: 338–344. 10.1111/j.1469-0691.2012.03806.x 22429458

[13] Chosidow O, Giraudeau B, Cottrell J, Izri A, Hofmann R, Mann SG, et al Oral ivermectin versus malathion lotion for difficult-to-treat head lice. N Engl J Med. 2010;362: 896–905. 10.1056/NEJMoa0905471 20220184

[14] Chaccour C, Hammann F, Rabinovich NR. Ivermectin to reduce malaria transmission I. Pharmacokinetic and pharmacodynamic considerations regarding efficacy and safety. Malar J. 2017;16: 161 10.1186/s12936-017-1801-4 28434401 PMC5402169

[15] Laing R, Gillan V, Devaney E. Ivermectin—Old Drug, New Tricks? Trends Parasitol. 2017;33: 463–472. 10.1016/j.pt.2017.02.004 28285851 PMC5446326

[16] Ōmura S, Crump A. Ivermectin and malaria control. Malar J. 2017;16 10.1186/s12936-017-1825-9 PMC5404691 28438169

[17] Leulmi H, Diatta G, Sokhna C, Rolain J-M, Raoult D. Assessment of oral ivermectin versus shampoo in the treatment of pediculosis (head lice infestation) in rural areas of Sine-Saloum, Senegal. Int J Antimicrob Agents. 2016;48: 627–632. 10.1016/j.ijantimicag.2016.07.014 27866866

[18] Mumcuoglu KY, Miller J, Rosen LJ, Galun R. Systemic activity of ivermectin on the human body louse (Anoplura: Pediculidae). J Med Entomol. 1990;27: 72–75. 2299658 10.1093/jmedent/27.1.72

[19] Pariser DM, Meinking TL, Bell M, Ryan WG. Topical 0.5% ivermectin lotion for treatment of head lice. N Engl J Med. 2012;367: 1687–1693. 10.1056/NEJMoa1200107 23113480

[20] Foucault C, Ranque S, Badiaga S, Rovery C, Raoult D, Brouqui P. Oral ivermectin in the treatment of body lice. J Infect Dis. 2006;193: 474–476. 10.1086/499279 16388498

[21] Sangaré AK, Rolain JM, Gaudart J, Weber P, Raoult D. Synergistic activity of antibiotics combined with ivermectin to kill body lice. Int J Antimicrob Agents. 2016;47: 217–223. 10.1016/j.ijantimicag.2016.01.001 26897755

[22] Chen L-P, Wang P, Sun Y-J, Wu Y-J. Direct interaction of avermectin with epidermal growth factor receptor mediates the penetration resistance in Drosophila larvae. Open Biol. 2016;6 10.1098/rsob.150231 PMC4852453 27249340

[23] Gao X, Yang J, Xu B, Xie W, Wang S, Zhang Y, et al Identification and Characterization of the Gene CYP340W1 from Plutella xylostella and Its Possible Involvement in Resistance to Abamectin. Int J Mol Sci. 2016;17 10.3390/ijms17030274 PMC4813138 26999122

[24] Diatta G, Abat C, Sokhna C, Tissot-Dupont H, Rolain J-M, Raoult D. Head lice probably resistant to ivermectin recovered from two rural girls in Dielmo, a village in Sine-Saloum, Senegal. Int J Antimicrob Agents. 2016;47: 501–502. 10.1016/j.ijantimicag.2016.03.013 27211825

[25] Kane NS, Hirschberg B, Qian S, Hunt D, Thomas B, Brochu R, et al Drug-resistant Drosophila indicate glutamate-gated chloride channels are targets for the antiparasitics nodulisporic acid and ivermectin. Proc Natl Acad Sci U S A. 2000;97: 13949–13954. 10.1073/pnas.240464697 11095718 PMC17681

[26] Luo L, Sun Y-J, Wu Y-J. Abamectin resistance in Drosophila is related to increased expression of P-glycoprotein via the dEGFR and dAkt pathways. Insect Biochem Mol Biol. 2013;43: 627–634. 10.1016/j.ibmb.2013.04.006 23648830

[27] Yoon KS, Strycharz JP, Baek JH, Sun W, Kim JH, Kang JS, et al Brief exposures of human body lice to sub-lethal amounts of ivermectin over transcribes detoxification genes involved in tolerance. Insect Mol Biol. 2011;20: 687–699. 10.1111/j.1365-2583.2011.01097.x 21895817 PMC3208734

[28] Trimbuch T, Rosenmund C. Should I stop or should I go? The role of complexin in neurotransmitter release. Nat Rev Neurosci. 2016;17: 118–125. 10.1038/nrn.2015.16 26806630

[29] Wang X, Wang R, Yang Y, Wu S, O’Reilly AO, Wu Y. A point mutation in the glutamate-gated chloride channel of Plutella xylostella is associated with resistance to abamectin. Insect Mol Biol. 2016;25: 116–125. 10.1111/imb.12204 26592158

[30] Dermauw W, Ilias A, Riga M, Tsagkarakou A, Grbić M, Tirry L, et al The cys-loop ligand-gated ion channel gene family of Tetranychus urticae: Implications for acaricide toxicology and a novel mutation associated with abamectin resistance. Insect Biochem Mol Biol. 2012;42: 455–465. 10.1016/j.ibmb.2012.03.002 22465149

[31] Wang D, Johnson AD, Papp AC, Kroetz DL, Sadée W. Multidrug resistance polypeptide 1 (MDR1, ABCB1) variant 3435C>T affects mRNA stability. Pharmacogenet Genomics. 2005;15: 693–704. 16141795

[32] Rebeiz M, Jikomes N, Kassner VA, Carroll SB. Evolutionary origin of a novel gene expression pattern through co-option of the latent activities of existing regulatory sequences. Proc Natl Acad Sci. 2011;108: 10036–10043. 10.1073/pnas.1105937108 21593416 PMC3121811

[33] Kimchi-Sarfaty C, Oh JM, Kim I-W, Sauna ZE, Calcagno AM, Ambudkar SV, et al A “silent” polymorphism in the MDR1 gene changes substrate specificity. Science. 2007;315: 525–528. 10.1126/science.1135308 17185560

[34] Gupta SK, Majumdar S, Bhattacharya TK, Ghosh TC. Studies on the relationships between the synonymous codon usage and protein secondary structural units. Biochem Biophys Res Commun. 2000;269: 692–696. 10.1006/bbrc.2000.2351 10720478

[35] Silva AX, Jander G, Samaniego H, Ramsey JS, Figueroa CC. Insecticide Resistance Mechanisms in the Green Peach Aphid Myzus persicae (Hemiptera: Aphididae) I: A Transcriptomic Survey. PLOS ONE. 2012;7: e36366 10.1371/journal.pone.0036366 22685538 PMC3369866

[36] Ahmad M, Denholm I, Bromilow RH. Delayed cuticular penetration and enhanced metabolism of deltamethrin in pyrethroid-resistant strains of Helicoverpa armigera from China and Pakistan. Pest Manag Sci. 2006;62: 805–810. 10.1002/ps.1225 16649192

[37] Long AA, Mahapatra CT, Woodruff EA, Rohrbough J, Leung H-T, Shino S, et al The nonsense-mediated decay pathway maintains synapse architecture and synaptic vesicle cycle efficacy. J Cell Sci. 2010;123: 3303–3315. 10.1242/jcs.069468 20826458 PMC2939802

[38] Casadio A, Longman D, Hug N, Delavaine L, Vallejos Baier R, Alonso CR, et al Identification and characterization of novel factors that act in the nonsense-mediated mRNA decay pathway in nematodes, flies and mammals. EMBO Rep. 2015;16: 71–78. 10.15252/embr.201439183 25452588 PMC4304730

[39] Abd El Halim HM, Alshukri BMH, Ahmad MS, Nakasu EYT, Awwad MH, Salama EM, et al RNAi-mediated knockdown of the voltage gated sodium ion channel TcNav causes mortality in Tribolium castaneum. Sci Rep. 2016;6: 29301 10.1038/srep29301 27411529 PMC4944135

[40] Pittendrigh BR, Olds BP, Yoon KS, Lee SH, Sun W, Clark JM. The genomics of human lice: From the genome to the potential for future control strategies. Pestic Biochem Physiol. 2013;106: 172–176. 10.1016/j.pestbp.2013.03.010

[41] Kwon DH, Kim JH, Kim YH, Yoon KS, Clark JM, Lee SH. Identification and characterization of an esterase involved in malathion resistance in the head louse Pediculus humanus capitis. Pestic Biochem Physiol. 2014;112: 13–18. 10.1016/j.pestbp.2014.05.006 24974112

[42] Huntwork S, Littleton JT. A complexin fusion clamp regulates spontaneous neurotransmitter release and synaptic growth. Nat Neurosci. 2007;10: 1235–1237. 10.1038/nn1980 17873870

[43] Forrester SG, Beech RN, Prichard RK. Agonist enhacement of macrocyclic lactone activity at a glutamate-gated chloride channel subunit from Haemonchus contortus. Biochem Pharmacol. 2004;67: 1019–1024. 10.1016/j.bcp.2003.08.047 15006538

[44] Pemberton DJ, Franks CJ, Walker RJ, Holden-Dye L. Characterization of Glutamate-Gated Chloride Channels in the Pharynx of Wild-Type and Mutant Caenorhabditis elegansDelineates the Role of the Subunit GluCl-α2 in the Function of the Native Receptor. Mol Pharmacol. 2001;59: 1037–1043. 10.1124/mol.59.5.1037 11306685

[45] Wolstenholme AJ, Rogers AT. Glutamate-gated chloride channels and the mode of action of the avermectin/milbemycin anthelmintics. Parasitology. 2005;131: S85–S95. 10.1017/S0031182005008218 16569295

[46] Kita T, Ozoe F, Azuma M, Ozoe Y. Differential distribution of glutamate- and GABA-gated chloride channels in the housefly Musca domestica. J Insect Physiol. 2013;59: 887–893. 10.1016/j.jinsphys.2013.06.005 23806605

[47] Probit Analysis. J Pharm Sci. 1971;60: 1432 10.1002/jps.2600600940

[48] Schmittgen TD, Livak KJ. Analyzing real-time PCR data by the comparative *C*_T_ method. Nat Protoc. 2008;3: nprot.2008.73. 10.1038/nprot.2008.73 18546601

