# Expression of Concern: Complexin in ivermectin resistance in body lice

**DOI:** 10.1371/journal.pgen.1010531

**Published:** 2022-12-13

**Authors:** The PLOS Genetics Editors

This article [1] has been identified as one of a series of submissions for which we have concerns about the reported research ethics approval information and the article’s adherence to PLOS research ethics policies.

PLOS will be investigating these concerns in accordance with COPE guidance and journal policies. Meanwhile, the *PLOS Genetics* Editors issue this Expression of Concern.

## References

[1] Amanzougaghene N, Fenollar F, Nappez C, Ben-Amara A, Decloquement P, Azza S, et al. (2018) Complexin in ivermectin resistance in body lice. PLoS Genet 14(8): e1007569. 10.1371/journal.pgen.1007569 30080859 PMC6108520

