# Correction: Complexin in ivermectin resistance in body lice

**DOI:** 10.1371/journal.pgen.1012258

**Published:** 2026-07-30

**Authors:** The PLOS Genetics Editors

This Correction resolves the prior Expression of Concern for the linked article [1,2].

Following the publication of the article and Expression of Concern [1,2], PLOS investigated the concerns pertaining to the reported ethical oversight and the article’s adherence to PLOS’ research ethics policies and PLOS’ Competing Interests policy.

Following the editorial investigation, the concerns pertaining to the article’s adherence to PLOS’ research ethics policies were dismissed.

The authors did not respond to the journal’s queries about competing interests. Based on publicly available information, the Editors update the article’s Competing Interests statement to: At the time this article was submitted, DR was listed as a co-founder and member of the scientific council of Arthrobac Pharma.

With this update, the *PLOS Genetics* Editors consider the issues described in [2] to be resolved. This Correction supersedes the prior Expression of Concern [2].
