# Supplementary figures and images for: Complexin in ivermectin resistance in body lice
Source: PLoS Genet. 2018 Aug 6;14(8):e1007569. doi: 10.1371/journal.pgen.1007569 (PMC6108520; doi:10.1371/journal.pgen.1007569)

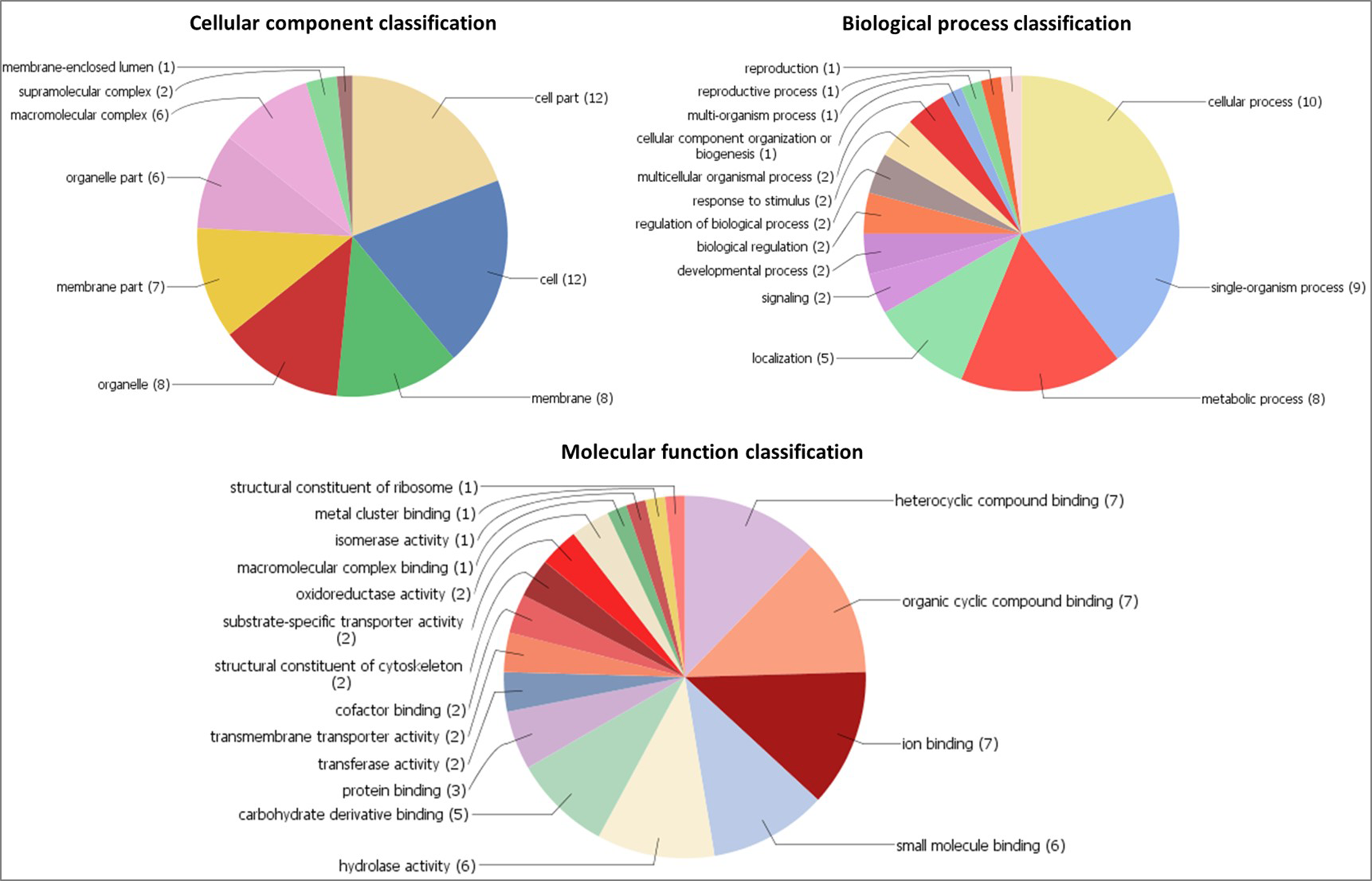

Supplement: S1 Fig — The proteins are grouped into three GO terms: cellular component, biological process and molecular function. (TIF) [file pgen.1007569.s001.tif]

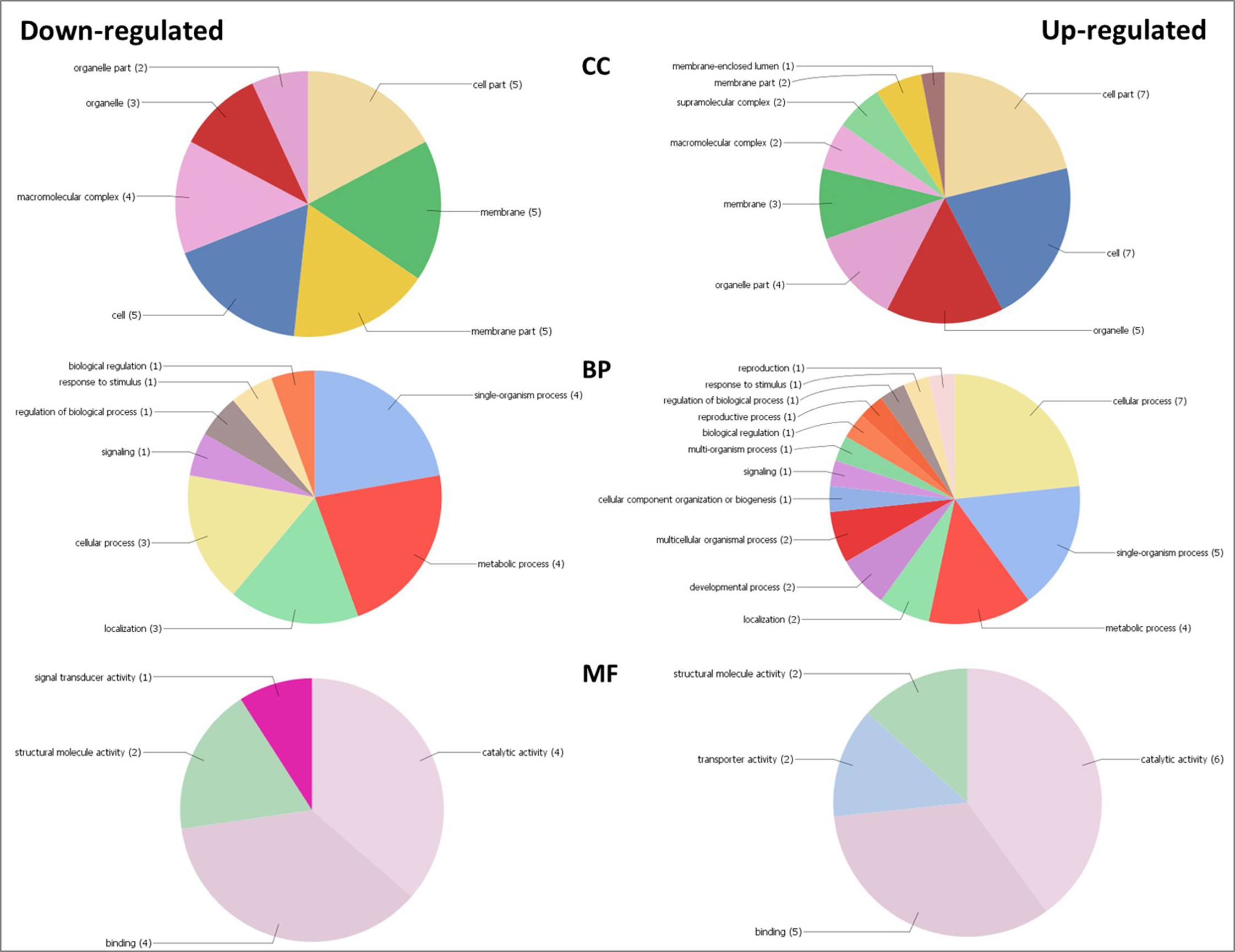

Supplement: S2 Fig — (TIF) [file pgen.1007569.s002.tif]

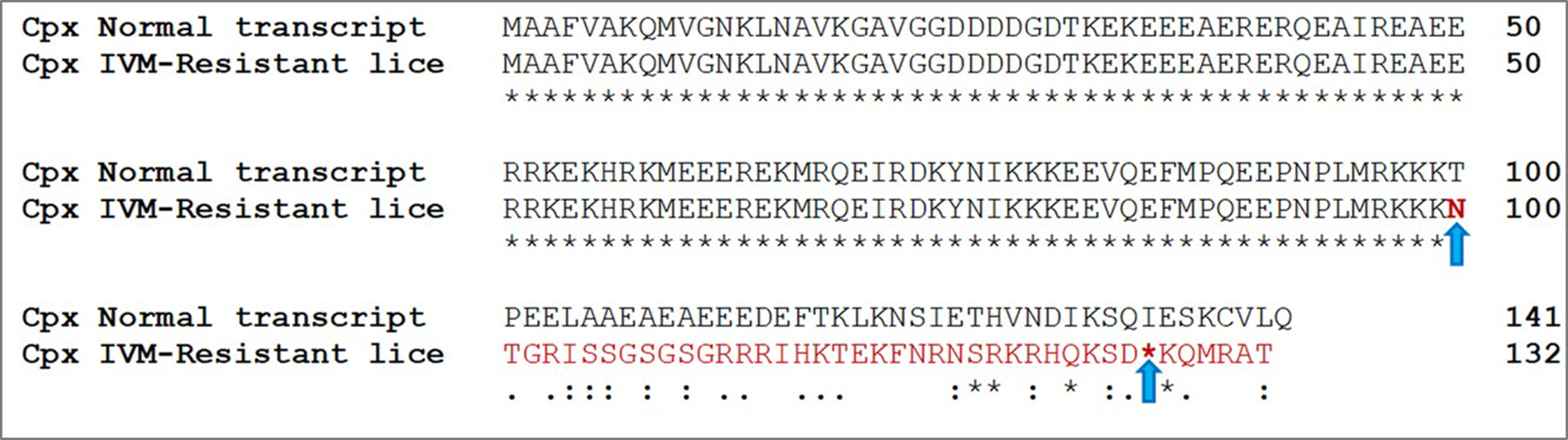

Supplement: S3 Fig — The alignment shows a frameshift starting at amino acid 100 and a premature stop codon at amino acid 111 in the mutated transcript (from the resistant lice) compared to normal Cpx transcript. (TIF) [file pgen.1007569.s003.tif]
