# Supplementary material for: Complexin in ivermectin resistance in body lice
Source: PLoS Genet. 2018 Aug 6;14(8):e1007569. doi: 10.1371/journal.pgen.1007569 (PMC6108520; doi:10.1371/journal.pgen.1007569)
Supplement: S1 Table — A total of 500 lice were exposed for each IVM dose. (DOC) [file pgen.1007569.s004.doc]

**S1 Table.**

| Time (hours) | Dose of ivermectin | | | | |
| --- | --- | --- | --- | --- | --- |
|  | 200 µg/kg | 150 µg/kg | 100 µg/kg | 75 µg/kg | 25 µg/kg |
| 0 | 500 | 500 | 500 | 500 | 500 |
| 12 | 265 | 372 | 495 | 480 | 494 |
| 24 | 85 | 280 | 436 | 438 | 478 |
| 48 | 2 | 105 | 385 | 391 | 470 |
| 72 | 0 | 44 | 302 | 330 | 464 |
| 96 | - | 4 | 250 | 327 | 444 |
| 120 | - | 0 | 159 | 262 | 389 |
| 144 | - | - | 128 | 244 | 375 |
