# Supplementary material for: Complexin in ivermectin resistance in body lice
Source: PLoS Genet. 2018 Aug 6;14(8):e1007569. doi: 10.1371/journal.pgen.1007569 (PMC6108520; doi:10.1371/journal.pgen.1007569)
Supplement: S2 Table — Probit regression data for the relationship between dose of ivermectin and mortality at 72 hours. (DOC) [file pgen.1007569.s005.doc]

**S2 Table.**

| Lice | LD_50_ (µg/kg) (95% CL) | LD_90_ (µg/kg) (95% CL) | Slope ± SE | χ2 |
| --- | --- | --- | --- | --- |
| S-Lab | 98.36 (83.56-114.67) 143,085 | 152.59 (132.76-187.76) | 3 ± 0.01 | 5.8 |

CL: Confidence limited; SE: Standard error; LD: Lethal dose
