# Supplementary material for: Complexin in ivermectin resistance in body lice
Source: PLoS Genet. 2018 Aug 6;14(8):e1007569. doi: 10.1371/journal.pgen.1007569 (PMC6108520; doi:10.1371/journal.pgen.1007569)
Supplement: S3 Table — (DOC) [file pgen.1007569.s006.doc]

**S3 Table.**

| **Amplification of primes** | **Gene name** | **Forward Sequence (3’-5’)** | **Reverse Sequence (3’-5’)** |
| --- | --- | --- | --- |
| **qPCR analysis** | Complexin | ACTGTAGCACGCATTTGCTTTC | GCCGCAAGAAGAACCAAAT |
|  | Trypsin | GCCGGATCATCCAAAGCTAA | TGAGTCCAACAACGGGTTTG |
|  | Clathrin heavy chain | GCCGTCAAAGCCGATAGAAC | CTTTCGGCAAATTCGTAGGC |
|  | 40S ribosomal | GGCGGTCGGTAAGAATAAGG | CCCTCGGATGCAATTTTTGT |
|  | Tubulinα1 | GAACTGTCGCCAAATTACTTCC | CGCTTGCTGTTTGTTATACAGG |
|  | Ornithine | CTCGGCAAATACGATTCTAGCT | CATCCCCGTATCGTAAACGTAT |
|  | Heat shock protein | CAAACCGTCGTACCAAGTGTTA | CTCACACATCGGAAACAACATC |
|  | Isocitrate dehydrogenase | AGGTGCTGG TGTTGTCGCG | ATTGAGCATTTTGGCGGCGC |
|  | Adenylate kinase | CTCGGTAACATCGGAGGA | GGGTGAAGACGCTGAAAAA |
|  | ATP synthase | GCTTCTGCAACTTCTACAGCAA | CCTGGAGTTGTAACCGTTTTTC |
|  | Heavy-chain filboin | ACCAAATCCAACAGCTGTACCT | CACCTTCCACAGGTGGATTT |
|  | Mitochondrial porin | GTGAGATTTTGACCTTCCGAAC | TCACATCAAGACAAGGGTTCAG |
|  | Limpet | CACAATATGGCTTCTCATCTCG | GACTCGTTGCGTCAAGTGTAAC |
|  | Tubulinß2 | TCCCTCTGCTTCTTTTCTAACG | GGTACAATGGATTCCGTACGTT |
|  | E0W0W7 | ACCGAGACCAGAACAAGATGT | CCGGAAAACAGTTGCAGTAAT |
|  | EF1α | CGTTTCCGTAAAAGAATTGCG | GGCTATGTGAGCCGTATGAC |
| **dsRNA** | Complexin | X.ATGCCGCAAGAAGAACCAA | X.TCACTGTAGCACGCATTT G |
|  | pQE30 | X.GTTCATCCATAGTTGCCTGACT | X.AGATAACACTGCGGCCAACTTAC |
| **RACE** | Complexin-GSP-5’ | CTTCCTCAGCTTCCGCTTCCGCTGC |  |
|  | Complexin-GSP-3’ | GCAGCGGAAGCGGAAGCTGAG |  |
| **Full-length amplification** | Complexin | ATGGCGGCTTTCGTTGCA | TCACTGTAGCACGCATTTGC |
|  | GluCl | ATGGAATACAGCGTTCAGCT | TTAATTATCTTCCGTATCTTCTC |

X: T7 promoter (TAATACGACTCACTATAGGG); GSP: Gene specific primer
