# Supplementary material for: Complexin in ivermectin resistance in body lice
Source: PLoS Genet. 2018 Aug 6;14(8):e1007569. doi: 10.1371/journal.pgen.1007569 (PMC6108520; doi:10.1371/journal.pgen.1007569)
Supplement: S4 Table — (DOC) [file pgen.1007569.s007.doc]

**S4 Table.**

| **Lice**  **(no. of exposed)** | **Time (hours)** | | | | | | | |
| --- | --- | --- | --- | --- | --- | --- | --- | --- |
|  | **0** | **12** | **24** | **48** | **72** | **96** | **120** | **144** |
| **Lab-IVR (300)** | 300 | 277 | 258 | 249 | 234 | 199 | 143 | 138 |
| **Lab-IVR (500)** | 500 | 372 | 280 | 105 | 44 | 4 | 0 | 0 |
