# Supplementary material for: Complexin in ivermectin resistance in body lice
Source: PLoS Genet. 2018 Aug 6;14(8):e1007569. doi: 10.1371/journal.pgen.1007569 (PMC6108520; doi:10.1371/journal.pgen.1007569)
Supplement: S5 Table — (DOC) [file pgen.1007569.s008.doc]

S5 Table.

| **GluCl mutation** | **Nucleotide mutation** | **Localization of mutation** | **Louse strain (Lab-IVR and Lab-IVS)** |
| --- | --- | --- | --- |
| **Mut-1** | T363C | N-terminal extracellular domain | Lab-IVR |
| **Mut-2** | T385C | N-terminal extracellular domain | Lab-IVR |
| **Mut-3** | G417A | N-terminal extracellular domain | Lab-IVR and Lab-IVS |
| **Mut-4** | G447A | N-terminal extracellular domain | Lab-IVR |
| **Mut-5** | A594G | M2 transmembrane domain | Lab-IVR |
| **Mut-6** | C897T | M3-M4 linker | Lab-IVR and Lab-IVS |
