# Supplementary material for: Complexin in ivermectin resistance in body lice
Source: PLoS Genet. 2018 Aug 6;14(8):e1007569. doi: 10.1371/journal.pgen.1007569 (PMC6108520; doi:10.1371/journal.pgen.1007569)
Supplement: S6 Table — (DOC) [file pgen.1007569.s009.doc]

**S6 Table.**

| **Gene** | **Gene abbreviation** | **FC_qRT-PCR_** | **FC_proteomic_** |
| --- | --- | --- | --- |
| Complexin | Cpx | -3.50 | -10.24 |
| Trypsinogen | Tryp | -2.02 | -3.80 |
| Clathrin heavy chain | CLTC | -3.12 | -3.24 |
| 40S ribosomal protein S3a | RPS3A | -1.82 | -3.93 |
| Tubulin α1 | TUBα1 | 3.61 | 3.05 |
| E0W0W7 | E0W0W7 | 3.01 | 3.85 |
| Ornithine aminotransferase | OAT | 4.77 | 2.20 |
| Heat shock protein | HSP | 2.69 | 2.03 |
| Isocitrate dehydrogenase subunit | Idh | 2.98 | 2.61 |
| Adenylate kinase | Adk | 3.17 | 3.08 |
| ATP synthase | ATPase | 3.39 | 2.23 |
| Heavy-chain filboin | Fib-H | 3.01 | 2.51 |
| Mitochondrial outer membrane porin channel | MO-porin | 4.39 | 2.12 |
| Limpet | Lmpt | 3.38 | 4.14 |
| Tubulin ß2 | Tubß2 | 1.89 | 2.31 |

FC: Fold change
