# Supplementary material for: Complexin in ivermectin resistance in body lice
Source: PLoS Genet. 2018 Aug 6;14(8):e1007569. doi: 10.1371/journal.pgen.1007569 (PMC6108520; doi:10.1371/journal.pgen.1007569)
Supplement: S7 Table — (DOC) [file pgen.1007569.s010.doc]

**S7 Table**.

| **Species list** | ***Cpx* Accession no.** |
| --- | --- |
| **Vertebrates** | |
| *Homo sapiens* | AHW56455 |
| *Narke japonica* | O42105 |
| *Rattus norvegicus* | NM_022864 |
| *Xenopus laevis* | NP_001087909 |
| *Bos taurus* | AAI22584 |
| **Nematodes** | |
| *Trichuris trichiura* | CDW57795 |
| *Strongyloides ratti* | CEF68549 |
| *Caenorhabditis elegans* | NP_490868 |
| **Arthropods** | |
| *Ixodes scapularis* | EEC07951 |
| *Daphnia pulex* | EFX87986 |
| *Aedes aegypti* | XP021712522 |
| *Drosophila melanogaster* | NM001170033 |
| *Cyphomyrmex costatus* | XP018396052 |
| *Anoplophora_glabripennis* | XM018709052 |
| *Stomoxys calcitrans* | XP013102718 |
| *Pediculus humanus* (this study) | **MG954374** |
