# Supplementary material for: Complexin in ivermectin resistance in body lice
Source: PLoS Genet. 2018 Aug 6;14(8):e1007569. doi: 10.1371/journal.pgen.1007569 (PMC6108520; doi:10.1371/journal.pgen.1007569)
Supplement: S8 Table — (DOC) [file pgen.1007569.s011.doc]

**S8 Table.**

| **Biological replicate** | **Target gene** | **Mean Ct** | **SD** | **SE** | **ΔCt** | **2^−ΔΔCt^** | **Mean ΔCt** | **SD** | **SE** | **Mean 2^−ΔΔCt^** |
| --- | --- | --- | --- | --- | --- | --- | --- | --- | --- | --- |
| 24h-test-1 | Cpx | 20.094 | 0.103 | 0.059 | 2.180 | 0.867 | 2.190 | 0.017 | 0.010 | **0.837** |
|  | EF1α | 17.914 | 0.602 | 0.348 |  |  |  |  |  |  |
| 24h-test-2 | Cpx | 20.327 | 0.065 | 0.038 | 2.181 | 0.793 |  |  |  |  |
|  | EF1α | 18.146 | 0.284 | 0.164 |  |  |  |  |  |  |
| 24h-test-3 | Cpx | 20.390 | 0.377 | 0.218 | 2.210 | 0.850 |  |  |  |  |
|  | EF1α | 18.181 | 0.064 | 0.037 |  |  |  |  |  |  |
| 24h-control-1 | Cpx | 19.258 | 0.339 | 0.452 | 1.974 | 1.000 | 1.932 | 0.074 | 0.042 | **1.000** |
|  | EF1α | 17.285 | 0.152 | 0.088 |  |  |  |  |  |  |
| 24h-control-2 | Cpx | 19.562 | 0.509 | 0.360 | 1.847 | 1.000 |  |  |  |  |
|  | EF1α | 17.715 | 0.089 | 0.052 |  |  |  |  |  |  |
| 24h-control-3 | Cpx | 19.259 | 0.430 | 0.304 | 1.975 | 1.000 |  |  |  |  |
|  | EF1α | 17.284 | 0.213 | 0.123 |  |  |  |  |  |  |
| 48h-test-1 | Cpx | 22.653 | 0.301 | 0.174 | 3.920 | 0.241 | 3.930 | 0.044 | 0.026 | **0.245** |
|  | EF1α | 18.733 | 0.070 | 0.041 |  |  |  |  |  |  |
| 48h-test-2 | Cpx | 22.519 | 0.107 | 0.062 | 3.892 | 0.254 |  |  |  |  |
|  | EF1α | 18.627 | 0.117 | 0.068 |  |  |  |  |  |  |
| 48h-test-3 | Cpx | 22.685 | 0.405 | 0.234 | 3.979 | 0.240 |  |  |  |  |
|  | EF1α | 18.707 | 0.172 | 0.100 |  |  |  |  |  |  |
| 48h-control-1 | Cpx | 19.050 | 0.063 | 0.044 | 1.865 | 1.000 | 1.899 | 0.030 | 0.017 | **1.000** |
|  | EF1α | 17.185 | 0.213 | 0.123 |  |  |  |  |  |  |
| 48h-control-2 | Cpx | 19.142 | 0.526 | 0.372 | 1.914 | 1.000 |  |  |  |  |
|  | EF1α | 17.228 | 0.189 | 0.109 |  |  |  |  |  |  |
| 48h-control-3 | Cpx | 19.065 | 0.230 | 0.162 | 1.920 | 1.000 |  |  |  |  |
|  | EF1α | 17.145 | 0.157 | 0.091 |  |  |  |  |  |  |
| 72h-test-1 | Cpx | 22.141 | 0.062 | 0.036 | 3.223 | 0.432 | 3.396 | 0.234 | 0.135 | **0.385** |
|  | EF1α | 18.918 | 0.233 | 0.135 |  |  |  |  |  |  |
| 72h-test-2 | Cpx | 21.744 | 0.141 | 0.082 | 3.303 | 0.388 |  |  |  |  |
|  | EF1α | 18.441 | 0.310 | 0.179 |  |  |  |  |  |  |
| 72h-test-3 | Cpx | 21.903 | 0.099 | 0.057 | 3.662 | 0.336 |  |  |  |  |
|  | EF1α | 18.241 | 0.287 | 0.166 |  |  |  |  |  |  |
| 72h-control-1 | Cpx | 19.506 | 0.531 | 0.375 | 2.013 | 1.000 | 2.013 | 0.077 | 0.044 | **1.000** |
|  | EF1α | 17.493 | 0.449 | 0.259 |  |  |  |  |  |  |
| 72h-control-2 | Cpx | 19.882 | 0.099 | 0.070 | 1.936 | 1.000 |  |  |  |  |
|  | EF1α | 17.946 | 0.178 | 0.013 |  |  |  |  |  |  |
| 72h-control-3 | Cpx | 19.267 | 0.020 | 0.014 | 2.089 | 1.000 |  |  |  |  |
|  | EF1α | 17.178 | 0.140 | 0.083 |  |  |  |  |  |  |

Test: Cpx dsRNA-injected; ontrol: pQE30 dsRNA-injected

For each biological replicate three technical replications were evaluated to calculate Mean Ct
