# Supplementary material for: Complexin in ivermectin resistance in body lice
Source: PLoS Genet. 2018 Aug 6;14(8):e1007569. doi: 10.1371/journal.pgen.1007569 (PMC6108520; doi:10.1371/journal.pgen.1007569)
Supplement: S9 Table — (DOC) [file pgen.1007569.s012.doc]

**S9 Table.**

| **Lice**  **(no. of exposed)** | **Time (hours)** | | | | | | | |
| --- | --- | --- | --- | --- | --- | --- | --- | --- |
|  | **0** | **12** | **24** | **48** | **72** | **96** | **120** | **144** |
| **Cpx dsRNA-injected (165)** | 165 | 129 | 116 | 104 | 88 | 70 | 44 | 39 |
| **pQE30 dsRNA-injected (150)** | 150 | 110 | 83 | 29 | 10 | 0 | - | - |
